# Improving Interoperability by Incorporating UnitsML Into Markup Languages

**DOI:** 10.6028/jres.115.003

**Published:** 2010-02-01

**Authors:** Ismet Celebi, Robert A. Dragoset, Karen J. Olsen, Reinhold Schaefer, Gary W. Kramer

**Affiliations:** Physics Laboratory, National Institute of Standards and Technology, Gaithersburg, MD, 20899; Wiesbaden Computer Integrated Laboratory (WICIL), RheinMain University of Applied Sciences, Wiesbaden, Germany; Physics Laboratory, National Institute of Standards and Technology, Gaithersburg, MD, 20899; Wiesbaden Computer Integrated Laboratory (WICIL), RheinMain University of Applied Sciences, Wiesbaden, Germany; Biochemical Science Division, National Institute of Standards and Technology, Gaithersburg, MD, 20899

**Keywords:** analytical experiments, AnIML, data storage, device integration, interoperability, Markup Language, Scientific Units of Measure, UnitsML, Web services, XML

## Abstract

Maintaining the integrity of analytical data over time is a challenge. Years ago, data were recorded on paper that was pasted directly into a laboratory notebook. The digital age has made maintaining the integrity of data harder. Nowadays, digitized analytical data are often separated from information about how the sample was collected and prepared for analysis and how the data were acquired. The data are stored on digital media, while the related information about the data may be written in a paper notebook or stored separately in other digital files. Sometimes the connection between this “scientific meta-data” and the analytical data is lost, rendering the spectrum or chromatogram useless. We have been working with ASTM Subcommittee E13.15 on Analytical Data to create the Analytical Information Markup Language or AnIML—a new way to interchange and store spectroscopy and chromatography data based on XML (Extensible Markup Language). XML is a language for describing what data are by enclosing them in computer-useable tags. Recording the units associated with the analytical data and metadata is an essential issue for any data representation scheme that must be addressed by all domain-specific markup languages. As scientific markup languages proliferate, it is very desirable to have a single scheme for handling units to facilitate moving information between different data domains.

At NIST, we have been developing a general markup language just for units that we call UnitsML. This presentation will describe how UnitsML is used and how it is being incorporated into AnIML.

## 1. Introduction

As scientific markup languages proliferate, it is very desirable to have a single scheme for handling scientific units of measure to facilitate moving information between different domains. Since units are independent of the software used, it is reasonable to separate units from the technical data. An incorrect description of a measurement unit can falsify an entire experiment. Therefore, it is important that the handling of units be appropriately developed to allow for the unambiguous storage, exchange, and processing of numeric data. Units of measure are not only needed by laboratory automation systems, but nearly all other application domains. Examples include: physics, chemistry, materials, and mathematics. The field of aeronautical and space engineering had the infamous Mars Climate Orbiter problem. The loss of NASA’s Climate Orbiter on September 23, 1999 was traced to a measurement unit problem. The 125 million dollar space orbiter was lost as it entered the orbit of Mars. Mission managers have concluded that the cause of the mishap was confusion over the type of units used to measure the strength of thruster firings. The problem was due to an error in communication between the Mars Climate Orbiter spacecraft team in Colorado and the mission navigation team in California. The peer review preliminary findings indicate that one team used English units (e.g., inches, feet, pounds) while the other used metric units for a key spacecraft operation [1, 2].

Developers have requested a single language for encoding units properties in XML. At the National Institute of Standards and Technology (NIST), we are developing a schema for encoding scientific units, quantities, and dimensions in XML, named UnitsML (Units Markup Language). The development and deployment of a markup language for units will allow for the unambiguous storage, exchange, and processing of numeric data, thus facilitating the collaboration and sharing of information. The usage of UnitsML in other markup languages will prevent duplication of effort and improve interoperability. Today there are many markup languages based on XML that could incorporate UnitsML including MathML (Mathematics Markup Language), AnIML (Analytical Information Markup Language), and AMDML (Atomic and Molecular Data Markup Language), etc.

## 2. Extensible Markup Language

XML (Extensible Markup Language) is a standard for the production of human and machine readable documents. XML is a W3C (World Wide Web Consortium)-recommended general-purpose markup language for creating special-purpose markup languages. A markup language is a mechanism to describe both markup and content in the same document. XML defines the rules for the syntax and structure of such documents. For a concrete XML application, the details of the respective documents must be specified. This requires the definition of structural components and their arrangement within a document tree. XML is therefore a standard for the definition of arbitrary markup languages. A markup language like XML, which is used for the definition of other languages, is called a meta language. One of the main purposes of XML is to facilitate the sharing of data across different systems or software modules or the sharing different types of data to be exported for interoperability or archival purposes [3–5].

## 3. Analytical Information Markup Language

Analytical Information Markup Language (AnIML), is a markup language for analytical chemistry data that is currently under development by ASTM subcommittee E13.15. It is a combination of a highly flexible core schema, a technique schema, and a set of analytical technique instance documents (ATID files). The core schema defines containers for result data in a generic manner. The ATID files are XML files, which apply tight constraints to the flexible core. Each ATID file refers to a specific analytical technique. The organisation of ATID files is specified by the technique schema. Extensions of ATID files are possible for vendor-specific, institutional-specific, and user-specific parameters. The goal of AnIML is to interchange and store analytical results and their meta data [6].

More information about AnIML can be found on the AnIML web site, http://www.animl.org/.

## 4. Units Markup Language

Units Markup Language (UnitsML) is a general XML-based markup language for encoding scientific units. It has a single schema for handling units, which is desirable to facilitate moving information between different data domains. The UnitsML schema is designed for incorporating scientific units into other XML documents or into any XML-based software. Various tools are under development to assist in the use of UnitsML.

“The value of a quantity is its magnitude expressed as the product of a number and a unit” [7]. The value of a quantity Q can be written as Q = N U, where N is the numerical value of Q when the value of Q is expressed in the unit U (Example: length = 5 m) [7]. UnitsML does not describe the numerical value; it only describes the unit.

The main requirement for use of UnitsML is the availability of its schema. It can be problematic for each user to collect information on units and the associated quantities and to define conversions to other units. Alternatively, users can refer to unit definitions from a third party database. Such a database containing information on units, prefixes, quantities, and dimensions encoded in the UnitsML schema is under development at NIST. This database, called UnitsDB, contains detailed units and dimensionality information for SI units and an extensive collection of common, non-SI units. The database includes information on units, quantities, symbols, language-specific unit names, and representations in terms of other units, including conversion factors to reference units. In the representations table, the units database describes all units in terms of the seven SI (International System of Units) base units [7]. In addition some units are described in terms of related, appropriate units. Table 1 shows the expression of farad in the database. Recall that a farad is a unit of capacitance equal to one coulomb per volt. Reducing the definition of farad to SI base units gives F = C · V^−1^ = m^−2^ · kg^−1^ · s^4^ · A^2^.

**Fig. 1:**
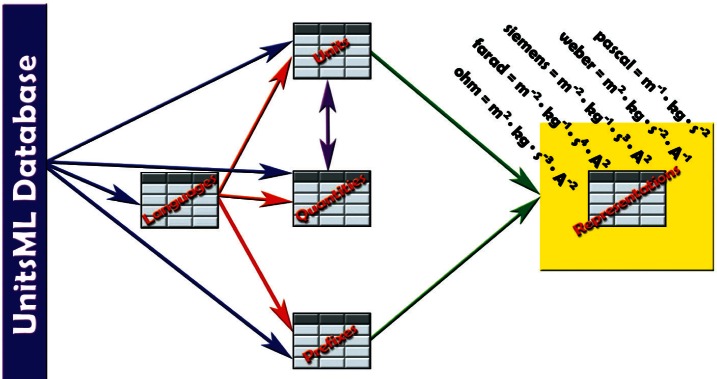
Storage of SI derived units in UnitsDB.

Figure 1 presents a few tables from UnitsDB and shows how SI-derived units are stored in the database.

More information about UnitsML can be found on the UnitsML website, http://unitsml.nist.gov/. More information about SI units can be found at http://www.bipm.org/ and http://physics.nist.gov/SP811/.

## 5. Ways to Incorporate UnitsML Into Other Markup Languages

UnitsML has been designed to be a component for inclusion into other markup languages. There are several different ways to incorporate UnitsML into other markup languages. These are referencing to the schema, including the schema, importing the schema, and redefining the schema elements.

### 5.1 Refer to the UnitsML Schema

UnitsML may be included in schema-based markup languages by referencing the UnitsML schema in an instance document. The W3C’s finalization of the XML Schema specification allows greater flexibility and specificity in defining constraints than are available with DTDs (Document Type Definitions). One important part of using schemas is being able to reference them within other XML documents. Making a reference from within an XML document requires a declaration of the XML schema instance namespace, a prefix mapping (xsi), and associated URI (Uniform Resource Identifier) to give access to the attributes needed for referencing the XML schemas. If needed, there can be defined a default namespace to provide a home for all non-prefixed elements in the document. Once the XML schema instance namespace is available, one can provide the schemaLocation attribute within it. The schemaLocation attribute consists of two values. The first value, or argument, is the namespace, which must be unique (URI), and the second is the actual resolvable schema location (URL—Uniform Resource Locator). In this case, the first referenced schema location is the host schema and the second the UnitsML schema. In the same way we could reference a third, fourth, or additional schemas. There are many more options for referencing schemas, using them with and without namespaces. These options are documented in the W3C XML Schema specification.

One way of incorporating UnitsML into AnIML documents by referencing is to create compound documents that reference the AnIML core schema and UnitsML schema. An example is shown in Listing 1.

Features of UnitsML can be incorporated into XML instance documents by using the actual UnitsML schema within the host schema. The problem with this is the availability of the UnitsML schema. The following methods are dependent on having the UnitsML schema file (.xsd). The user could download the UnitsML schema to make it available offline. In this case, the user is responsible for updating the UnitsML schema, when schema updates are available on the UnitsML server. The UnitsML tool, which is described below in “Tools under development,” should be able to warn the user of this update and to update the offline schema. To do this some changes must be made in the host schemas. There are three ways that this can be carried out:

### 5.2 <include> the UnitsML Schema

This directive results in the UnitsML schema being brought into the host schema within the host schema namespace. The element <include> brings in definitions and declarations from the UnitsML schema into the host schema. It requires the UnitsML schema to be in the same target namespace as the host schema namespace [8].

<xs:include schemaLocation = "unitsml.xsd"/>

Listing 2 shows an example of the include method on an AnIML instance document. Compared with the import example shown in Listing 3, we see the difference in namespaces.

**Listing 1.**
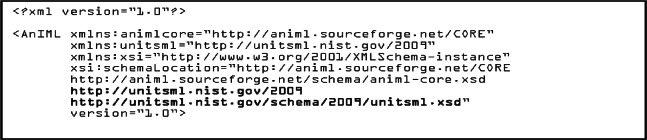
AnIML Core with UnitsML Schema-Referencing.

**Listing 2.**
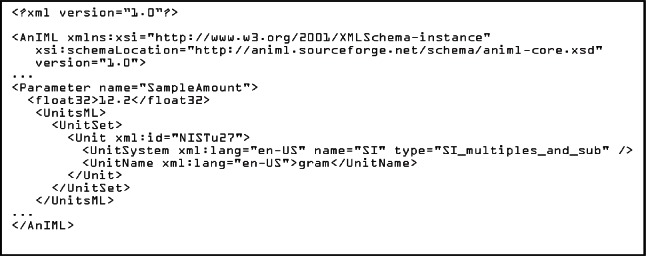
AnIML Core with UnitsML included in the schema.

### 5.3 <import> the UnitsML Schema

The import function behaves similarly to the include directive with the difference that it is possible to import elements from other namespaces. In the example below, only the units element is imported from the UnitsML schema [8].

<xs:import namespace="http://unitsml.nist.gov/2009"
schemaLocation="unitsml.xsd"/>
<xs:element ref="unitsml:units"/>

Using the import option, an AnIML data file would look like the example shown in Listing 3. It shows that the AnIML core namespace (xmlns:animlcore) is different than the UnitsML namespace (xmlns:unitsml) and that the units part of the document is described completely in UnitsML. The following element of the <UnitSet> element <Unit> is defined globally in the UnitsML schema. Therefore since this example doesn’t need information on prefixes, quantities, or dimensions, it is possible to use the <Unit> element directly without using the root element <UnitsML>.

### 5.4 <redefine> the Elements of UnitsML

The redefine directive can be used in place of the include function. This directive, however, allows elements from the UnitsML schema to be redefined to meet current needs in the combined schema [8].

<xs:redefine schemaLocation="unitsml.xsd">

The redefined elements from the UnitsML schema are placed here.

</xs:redefine>

The instance documents using redefined schema elements look the same as those using the include method. An example is given in Listing 2.

**Fig. 2:**
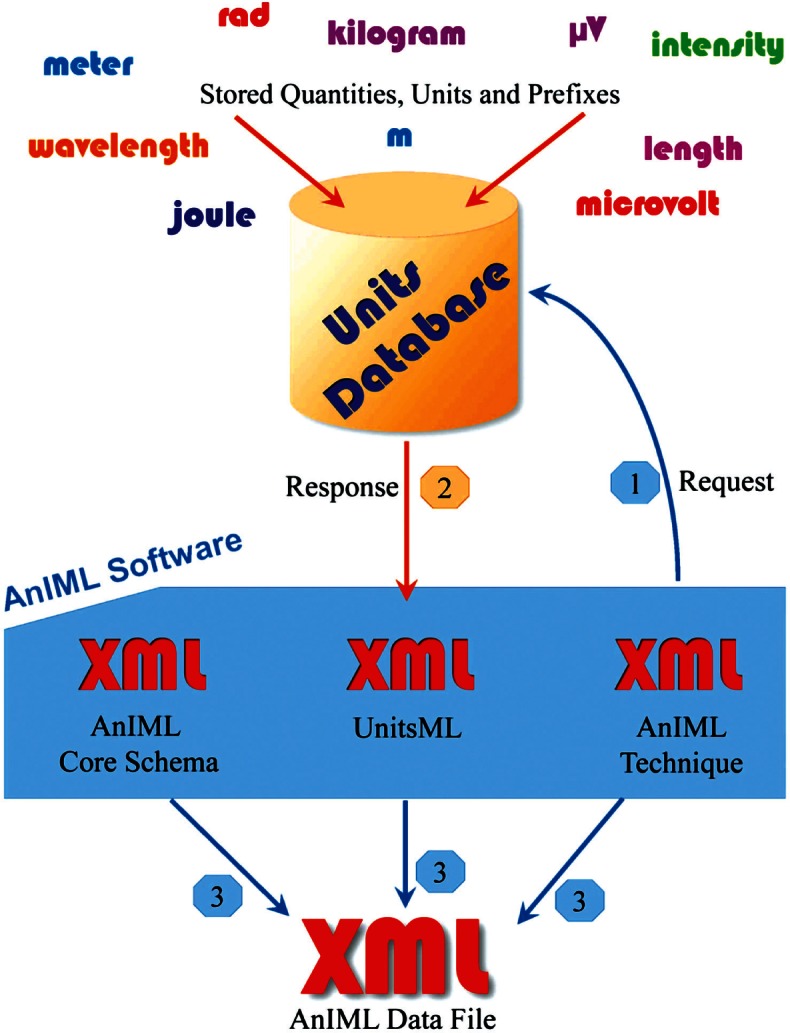
Structural overview of incorporating UnitsML into a compound data file. The event sequence is: 1. request; 2. response; 3. generating instance document.

AnIML is a little different than other markup languages because AnIML works with two schemas. It has a core and a technique schema. In this case there are actually three schemas, including the UnitsML schema. Figure 2 shows one possible method of incorporating UnitsML into AnIML. This example requires that the AnIML client have real-time access to the internet to get the information from the UnitsDB database.

Table 2 summarizes the four options for incorporating UnitsML into a host markup language.

**Listing 3.**
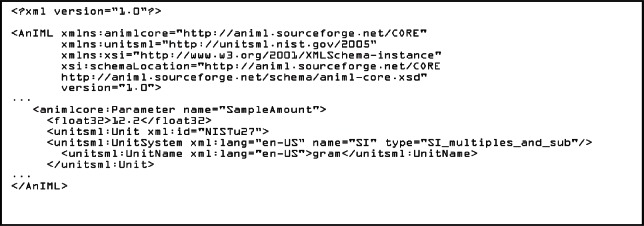
AnIML Core with UnitsML imported in the schema.

## 6. Tools Under Development

**Fig. 3:**
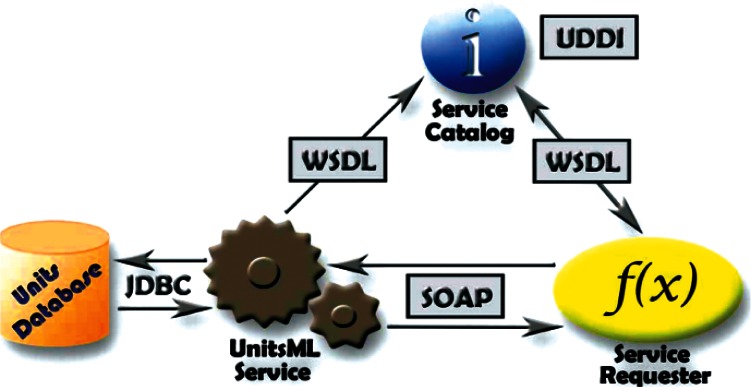
UnitsML Web Service.

We are currently working on web services to process queries that will return UnitsML code containing information from the UnitsDB. A web service provides integration over existing internet protocols, which makes the service compatible with most operating systems and programming languages. To use the web service, clients are required to support the XML-based Web Service Description Language (WDSL) and the XML-based exchange protocol SOAP (formerly Simple Object Access Protocol). Most recently developed web services packages support these standards. Figure 3 shows how the UnitsML web services will work. The service information could be published using the XML-based UDDI (Universal Description, Discovery, and Integration) protocol. Applications can look up web services information to determine options to use. The public interface to the web service is described by the WSDL, an XML-based service description on how to communicate using the web service. After the client receives the information describing the services, the communication between client and server uses the SOAP protocol. The services in the UnitsML Server will be written in Java and will use the JDBC (Java Database Connectivity) driver to communicate with the database. The internal processing of the XML file in the UnitsML Server will be done using XML tools such as, a data binding framework, SAX (Simple API for XML), and DOM (Document Object Model) [3–5].

We are also working on a solution to manage offline-stored units information in UnitsML for clients lacking a real-time internet connection. With this tool, users will be able to manage their own copies of UnitsML data and will not be constantly dependent on access to UnitsDB. The ability to edit and view available unit information without specific XML knowledge will make the use of UnitsML easier. The ability of the tool to connect to the UnitsML web services and update the offline available unit information is intended.

Development of the UnitsML schema has initially taken place at NIST, but completion of the development process should also include input from the international scientific and engineering community. To this end, an OASIS Technical Committee has been created to address any needed changes in the schema and to publish a final recommendation for UnitsML. The release data for UnitsDB and the Web Services tool will be sometime after the recommendation for the UnitsML schema has been published.

## Figures and Tables

**Table 1:** Storage of the unit farad in UnitsDB

Base Unit	Prefix	Power Numerator
meter	none	−2
kilogram	none	−1
second	none	4
ampere	none	2

**Table 2:** Overview of the ways to incorporate UnitsML into host markup language

Incorporation Method	Reference	Include	Import	Redefine
Different Namespace option	Yes	No	Yes	No
Redefine of elements option	No	No	No	Yes
Changes in host schema required	No	Yes	Yes	Yes
